# Description of 3,180 Courses of Chelation with Dimercaptosuccinic Acid in Children ≤5 y with Severe Lead Poisoning in Zamfara, Northern Nigeria: A Retrospective Analysis of Programme Data

**DOI:** 10.1371/journal.pmed.1001739

**Published:** 2014-10-07

**Authors:** Natalie Thurtle, Jane Greig, Lauren Cooney, Yona Amitai, Cono Ariti, Mary Jean Brown, Michael J. Kosnett, Krystel Moussally, Nasir Sani-Gwarzo, Henry Akpan, Leslie Shanks, Paul I. Dargan

**Affiliations:** 1 Médecins Sans Frontières, Amsterdam, Holland; 2 Médecins Sans Frontières, London, United Kingdom; 3 Department of Management, Bar Ilan University, Ramat Gan, Israel; 4 London School of Hygiene & Tropical Medicine, London, United Kingdom; 5 Healthy Homes/Lead Poisoning Prevention Program, National Center for Environmental Health, Centers for Disease Control and Prevention, Atlanta, Georgia, United States of America; 6 Division of Clinical Pharmacology and Toxicology, Department of Medicine, University of Colorado School of Medicine, Denver, Colorado, United States of America; 7 Department of Public Health, Federal Ministry of Health, Abuja, Nigeria; 8 Federal Ministry of Health, Abuja, Nigeria; 9 Federal Ministry of Communication Technology, Abuja, Nigeria; 10 Guy's and St. Thomas' NHS Foundation Trust, London, United Kingdom; Simon Fraser University, Canada

## Abstract

**Background:**

In 2010, Médecins Sans Frontières (MSF) discovered extensive lead poisoning impacting several thousand children in rural northern Nigeria. An estimated 400 fatalities had occurred over 3 mo. The US Centers for Disease Control and Prevention (CDC) confirmed widespread contamination from lead-rich ore being processed for gold, and environmental management was begun. MSF commenced a medical management programme that included treatment with the oral chelating agent 2,3-dimercaptosuccinic acid (DMSA, succimer). Here we describe and evaluate the changes in venous blood lead level (VBLL) associated with DMSA treatment in the largest cohort of children ≤5 y of age with severe paediatric lead intoxication reported to date to our knowledge.

**Methods and Findings:**

In a retrospective analysis of programme data, we describe change in VBLL after DMSA treatment courses in a cohort of 1,156 children ≤5 y of age who underwent between one and 15 courses of chelation treatment. Courses of DMSA of 19 or 28 d duration administered to children with VBLL ≥ 45 µg/dl were included. Impact of DMSA was calculated as end-course VBLL as a percentage of pre-course VBLL (ECP). Mixed model regression with nested random effects was used to evaluate the relative associations of covariates with ECP. Of 3,180 treatment courses administered, 36% and 6% of courses commenced with VBLL ≥ 80 µg/dl and ≥ 120 µg/dl, respectively. Overall mean ECP was 74.5% (95% CI 69.7%–79.7%); among 159 inpatient courses, ECP was 47.7% (95% CI 39.7%–57.3%). ECP after 19-d courses (*n = *2,262) was lower in older children, first-ever courses, courses with a longer interval since a previous course, courses with more directly observed doses, and courses with higher pre-course VBLLs. Low haemoglobin was associated with higher ECP. Twenty children aged ≤5 y who commenced chelation died during the period studied, with lead poisoning a primary factor in six deaths. Monitoring of alanine transaminase (ALT), creatinine, and full blood count revealed moderate ALT elevation in <2.5% of courses. No clinically severe adverse drug effects were observed, and no laboratory findings required discontinuation of treatment. Limitations include that this was a retrospective analysis of clinical data, and unmeasured variables related to environmental exposures could not be accounted for.

**Conclusions:**

Oral DMSA was a pharmacodynamically effective chelating agent for the treatment of severe childhood lead poisoning in a resource-limited setting. Re-exposure to lead, despite efforts to remediate the environment, and non-adherence may have influenced the impact of outpatient treatment.

*Please see later in the article for the Editors' Summary*

## Abstract 2

Jane Greig and colleagues from the medical humanitarian organization Médecins Sans Frontières describe the use of the oral chelating agent dimercaptosuccinic acid (DMSA) in several thousand young children with severe lead poisoning as a result of an environmental disaster in Zamfara, northern Nigeria.

*Please see later in the article for the Editors' Summary*

## Editors' Summary

**Background:**

Lead, a toxic metal that occurs naturally in the earth's crust, is now present throughout the environment because of human activities. For many years, lead was added to paint and gasoline and used in solder for water pipes. In addition, the mining, smelting, and refining of some metallic ores releases lead into the environment. Inhalation of contaminated air, consumption of contaminated food and water, and contact with dust that contains lead raises venous blood lead levels (VBLLs) and causes many health problems, particularly in children. Children who ingest large amounts of lead can develop anemia, muscle weakness, kidney damage, and life-threatening encephalopathy (brain swelling). Although fatal lead poisoning is now rare in resource-rich countries, it nevertheless remains a major global health problem. Over a three-month period in early 2010, for example, about 400 young children died in Zamfara State, Nigeria, from unexplained, intractable fits. By May 2010, it was clear that recently expanded gold mining had caused widespread environmental lead contamination in the region, and an environmental management program was begun to reduce lead levels in the surface soils.

**Why Was This Study Done?:**

In response to the lead poisoning outbreak, the not-for-profit organization Médecins Sans Frontières (MSF) began a medical management program to reduce VBLLs that included treatment with the oral chelation agent dimercaptosuccinic acid (DMSA). Chelation agents bind metal ions and facilitate their removal from the body, thereby reducing the likelihood of lead moving from the blood to the brain. Lead encephalopathy has been commonly treated by injecting another chelator called CaNa_2_EDTA, but the discovery of more than 1,000 cases of childhood lead poisoning in rural villages in Nigeria meant that MSF needed a chelation approach that could be applied rapidly in a remote resource-limited setting. Additionally, although CaNa_2_EDTA has been in common use for severe lead poisoning for longer than DMSA, and is commonly recommended in guidelines, the evidence base does not support one treatment as superior. Here, in a retrospective analysis of MSF program data, the researchers evaluate the changes in VBLLs before and after courses of oral DMSA treatment in children aged five years and below living in Zamfara to gain new insights into this understudied treatment for severe childhood lead poisoning.

**What Did the Researchers Do and Find?:**

The researchers measured VBLLs before and after treatment with DMSA in 1,156 children (inpatient and outpatient) with high amounts of lead in their blood who underwent one or more courses of chelation treatment lasting 19 or 28 days by calculating each child's end-course VBLL as a percentage of the child's pre-course VBLL (ECP). Considering all the treatment courses given between June 2010 and June 2011, the mean (average) ECP was 74.5%. That is, on average, VBLLs measured at the end of treatment courses were reduced by a quarter compared to VBLLs at the start of treatment courses. Among 159 inpatient courses of DMSA, the ECP was 47.7% (a halving of pre-course VBLLs). The ECP after 19-day courses was lower in older children, after first-ever courses, after courses with a longer interval since a previous course, after courses that included more directly observed doses (DMSA given in the presence of a health-care worker), and in children with higher pre-course VBLLs. Nine of the children included in this analysis died during the study period; lead poisoning was probably involved in three of these deaths. Importantly, no clinically severe adverse effects related to DMSA were seen during the study period, and no laboratory findings were recorded that required treatment discontinuation.

**What Do These Findings Mean?:**

Because many changes were made to the treatment given to the affected children in Zamfara during the study period and because no information is presented here on clinical outcomes, these findings cannot be used to reach any definitive conclusions about the effectiveness or safety of oral DMSA as a treatment for lead poisoning in young children. However, these findings show that chelation was associated with a large reduction in the death rate among probable or suspected cases of childhood lead poisoning in Zamfara and provide new information about oral chelation that may help agencies such as MSF provide urgent treatment for lead poisoning in resource-limited settings where intravenous chelation is not feasible. Moreover, the finding of a lower ECP after inpatient treatment courses compared to after outpatient courses suggests that re-exposure to lead and non-adherence to treatment may have influenced the impact of outpatient treatments. Thus, it is essential that medical management of lead poisoning in resource-limited settings be accompanied by environmental remediation and that efforts are made to support adherence to treatment in the community by implementing directly observed treatment wherever possible.

**Additional Information:**

Please access these websites via the online version of this summary at http://dx.doi.org/10.1371/journal.pmed.1001739.

- A related *PLOS ONE* Research Article by Greig et al. provides information about the association between VBLLs and neurological features in children affected by the acute lead poisoning outbreak in Zamfara
- MSF provides information about the lead poisoning crisis in Zamfara State
- Human Rights Watch, an international organization that works to uphold human dignity and advance the cause of human rights for all, also provides information about lead poisoning in Zamfara State, including photographs and a video
- Tox Town, an interactive site about environmental health concerns from the US National Library of Medicine, provides information on exposure to lead (in English and Spanish)
- The US Environmental Protection Agency provides information on lead and lead poisoning (in English and Spanish)
- The US Centers for Disease Control and Prevention provides information about lead in the environment and about its lead poisoning prevention program
- MedlinePlus provides a list of links to further information about lead poisoning (in English and Spanish)

## Introduction

Lead poisoning is a major and likely under-reported global health problem. There are many widely prevalent sources of lead exposure: lead-acid battery maanufacturing and recycling [1]; mining, smelting, and refining of lead and other ores [2]; traditional medicines [3]; residential paint [4]; ceramic glaze and artisanal pottery [5]; ammunition; and metal alloys or pigments used in the fabrication or coating of children's toys and jewellery. Most of these sources are poorly regulated in resource-poor countries.

Lead poisoning causes a continuum of effects ranging from subtle deficits in neurocognitive function to hypertension, cardiovascular disease, anaemia [6], renal dysfunction [7], and acute life-threatening encephalopathy [8],[9]. Lead-related child deaths in US cities in the 1950s and 1960s were frequent [10],[11] and were primarily related to lead paint ingestion. Chisolm and Harrison reported 36 cases of severe lead encephalopathy between 1954 and 1956 [12], and 38 cases were reported by Greengard et al. between 1959 and 1963 [13]. Fatal lead poisoning in resource-rich countries has rarely been reported in recent decades. The last death directly attributed to lead toxicity in the US occurred in 2006 in a child who swallowed a metallic charm [14]. However, in resource-poor countries, outbreaks are being reported with increasing frequency. An outbreak in Senegal in 2007 associated with informal lead-acid battery recycling resulted in a mean venous blood lead level (VBLL) of 130 µg/dl in 50 children, with at least 18 deaths [1]. In China, environmental exposure to industrial emissions has been linked to lead poisoning—though with comparatively low lead levels [15] and with no detailed reports of encephalopathic children.

In March 2010, the medical humanitarian aid organisation Médecins Sans Frontières (MSF) responded to reports of unusual mortality in children ≤5 y old in Zamfara State, northern Nigeria [16],[17]. Over a 2-mo period until 17 May 2010, nearly 300 children aged ≤5 y presented in four villages with intractable seizures of unknown aetiology, with a mortality of 48%. Before this period, many other children were reported to have died; the total number of deaths has been estimated at around 400. Recently expanded artisanal gold mining, using rudimentary processing techniques with ore that was heavily lead-laden, had resulted in the release of large amounts of lead dust. In May 2010, MSF and the US Centers for Disease Control and Prevention (CDC) identified widespread environmental lead contamination. This was fully characterised by TerraGraphics Environmental Engineering in conjunction with other agencies, which recorded surface soil concentrations within the affected villages of >10% lead (>100,000 ppm) [18]. The screening level set by the US Environmental Protection Agency above which remediation should usually occur in areas where children play is 400 ppm [19]. TerraGraphics Environmental Engineering, the Zamfara state government, and the Blacksmith Institute initiated environmental remediation after mining activities were moved some kilometres from village borders. Health promotion addressed ways to prevent further lead exposure.

Although the therapeutic efficacy of lead chelation in reducing morbidity and mortality has never been demonstrated in a randomised controlled trial [20], a net overall reduction in blood lead is thought to be beneficial in severe intoxication by decreasing the potential for ongoing translocation of lead from blood to brain. MSF started a lead poisoning treatment programme using dimercaptosuccinic acid (DMSA, succimer) chelation in June 2010. By the end of 2012, MSF had screened 2,912 children aged ≤5 y in seven remediated villages; 94% of the children had VBLLs above 10 µg/dl (range 3–708) [16],[19]. The programme is ongoing, and more than 15,000 treatment courses have been commenced for children, with up to 34 courses per child—this paper examines data from the first year of the response.

Chelating agents used to treat lead poisoning include intravenous (IV) calcium disodium versenate (CaNa_2_EDTA), intramuscular dimercaprol, and oral DMSA. DMSA, a less toxic, water-soluble analogue of dimercaprol, was first introduced as a possible antidote to lead poisoning in the late 1950s [21],[22],[23]. In several studies, DMSA has been observed to be an effective oral chelator of lead with a low incidence of major adverse events [24],[25],[26],[27],[28]. It has typically not been used as a sole pharmaceutical agent in patients with overt lead encephalopathy because of theoretical concerns that it might have less utility than parenteral CaNa_2_EDTA in reducing high VBLL [20],[25]. However, the only comprehensive review available of CaNa_2_EDTA versus DMSA did not find evidence for superiority of one chelator over the other, and concluded that DMSA could be considered in circumstances where oral therapy is preferable [29].

Although removal from lead exposure remains the single most important intervention for the overexposed child and will usually be followed by a decline in blood lead, the accelerated reduction induced by chelation is recommended at VBLL ≥ 45 µg/dl to avert potential progression to encephalopathy [30],[31]. DMSA has been the recommended first-line chelation agent for non-encephalopathic patients with VBLL ≥ 45 µg/dl since 1995 [31]. While there are regimen recommendations, there remains some uncertainty regarding optimal dosing, length of course, length of time between courses, and adverse effects, particularly in severe poisoning, as reports of treatment of sizeable cohorts of severely lead-poisoned children are rare [24],[32]. In the US in 2010, only 347 (0.009%) of almost 4 million children ≤5 y screened had VBLL ≥ 45 µg/dl, and of these only 51 (0.001%) had VBLL ≥ 70 µg/dl [33]. CaNa_2_EDTA has generally been recommended as the first-line regimen for children with VBLL > 70 µg/dl, and published reports of the use of DMSA in children with VBLL > 100 µg/dl are limited [34],[35]. The sudden discovery of more than 1,000 active cases of moderate and severe childhood lead poisoning in rural villages in northern Nigeria required an approach to chelation suitable for this remote, resource-limited setting where safe, sterile administration of individualised dosages of IV CaNa_2_EDTA was not feasible. DMSA alone was therefore used, given orally in conscious children and via nasogastric tube in encephalopathic patients. Once the project was well-established, IV CaNa_2_EDTA was administered to encephalopathic children.

In a retrospective analysis of clinical data from a cohort of more than 1,000 lead-poisoned children in rural Zamfara, Nigeria, we evaluated the changes in VBLL after oral chelation treatment with DMSA and the occurrence of adverse drug effects associated with DMSA treatment. Using regression models in the largest such cohort of chelated children to our knowledge reported to date, we examined the relative associations of different dose regimens and other covariates with the magnitude of blood lead reduction achieved by a course of chelation treatment.

## Methods

### Ethics Statement

This study met the standards set by the independent MSF Ethics Review Board for retrospective analyses of routinely collected programmatic data [36]. These standards include, but are not limited to, assurances of confidentiality, involvement of local partners, and minimal harm to patients. Caregivers were informed that routine data were being collected for clinical care and could be used anonymised for programme monitoring and increasing knowledge about lead poisoning. Coded identification numbers were used, and personal identifiers and all unnecessary data were removed from the dataset. Review of anonymous, routinely collected programmatic data does not constitute research under the Nigerian National Health Research Ethics Committee guidelines. CDC involvement did not require Human Subjects Review as CDC personnel were not involved in treating patients.

### Chelation Protocol

The DMSA protocol in Zamfara aimed to deliver effective chelation that was acceptable to the community and appropriate to the challenging logistical context. If initial VBLL was ≥ 45 µg/dl, children were treated with oral DMSA (Chemet, Lundbeck; Succicaptal, Serb Laboratories). The contents of the DMSA capsules were mixed with Plumpy’Nut (therapeutic food paste, Nutriset) or milk for infants, or sprinkled on honey for older children. For obtunded encephalopathic children, the contents of the capsules were mixed with water, and the slurry was administered by nasogastric tube (*n = *14/3,180 [0.44%] chelation courses included in analysis of end-course VBLL as a percentage of pre-course VBLL [ECP]; *n = *12/2,000 [0.6%] courses excluded from ECP analysis).

**Figure 1:**
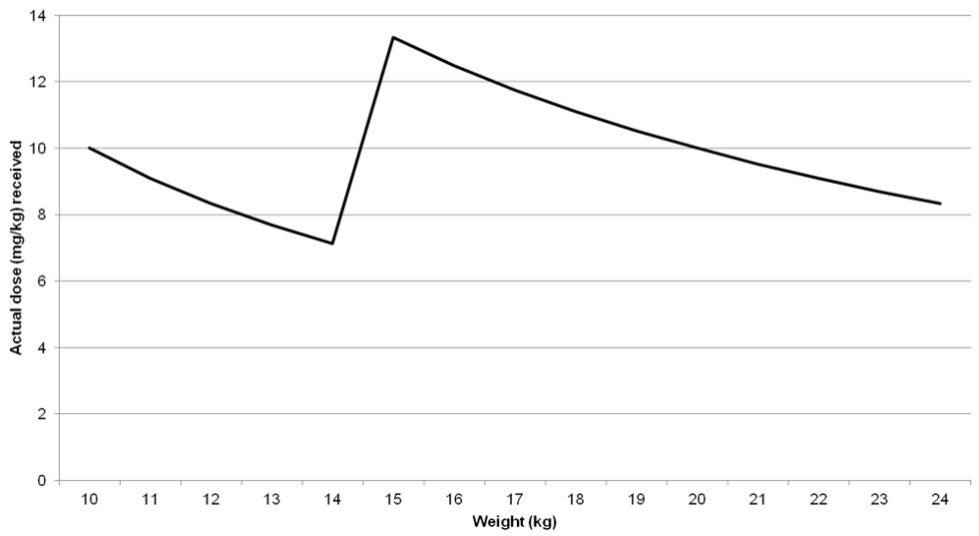
Representation of actual DMSA dose received by weight with rounding of 100-mg tablets.

Children were initially treated in June and July 2010 with 28-d DMSA 10 mg/kg twice daily (BD), based on the protocol used during the WHO- and CDC-supported intervention for a lead poisoning outbreak in Kosovo [2],[37]. Subsequently, most patients were chelated using repeated 19-d DMSA treatment schedules based on the US Food and Drug Administration's approved drug labelling. Each dose of DMSA was rounded to 100 mg (patient weight <15 kg) or 200 mg (15–24 kg) for ease of administration (Figure 1). Because of the severity of their lead intoxication, children with pre-course VBLL ≥ 120 µg/dl were treated with 10 mg/kg thrice daily (TDS) for the full 19 d. A minimum of 2 wk between courses is recommended unless blood lead levels indicate the need for more prompt treatment [35], so the VBLL measured at the end of each course determined the timing of the next course or VBLL test. All children received identical weight-based supplementation with zinc sulphate, calcium carbonate, iron sulphate, and folic acid (Table 1). Several minor revisions of the protocol that streamlined the treatment and blood test regimen and minimised burdens on patients and caretakers were undertaken following an MSF expert advisory group's interim analysis of blood lead trends during courses of chelation. Box 1 describes the revision process, Box 2 summarises key revisions, and Table 2 gives protocol details. All of the DMSA regimens have been used previously in clinical trials and clinical toxicology practice [26],[27],[38],[39],[40].

**Table 1:** Supplements to be provided throughout the chelation course and 2 wk after ceasing treatment.

Supplement	Recipient	Quantity
**Vitamin C**	**All children**	250 mg BD
**Calcium (calcium carbonate)**	**<1 y**	250 mg OD
	**1–3 y**	500 mg OD
	**4–8 y**	750 mg OD
**Multivitamin**	**All children**	1 tab OD
**Zinc (zinc sulphate)**	**<10 kg**	5 mg OD
	**10–25 kg**	10 mg OD
	**>25 kg**	20 mg OD
**Iron (tablets of ferrous sulphate 60 mg + folic acid 0.4 mg)**	**Hb > 120 g/l**	
	<16 kg	¼ tab OD
	16–30 kg	½ tab OD
	>30 kg	1 tab OD
	**Hb 110–120 g/l**	
	<16 kg	½ tab OD
	16–20 kg	1 tab OD
	>20 kg	1½ tab OD
	Hb < 110* g/l	
	<12 kg	½ tab OD
	12–20 kg	1 tab OD
	>20 kg	1½ tab OD

*Dose halved for first 2 d.

OD, once daily; tab, tablet.

**Table 2:** Developments in chelation protocol.

Development	Protocol Revision Number				
	1	2	3	4	5*
**Date commenced**	01 Jun 2010	10 Jul 2010	01 Oct 2010	12 Nov 2010	29 Mar 2011
**VBLL at which treatment starts**	≥45	≥45	≥45	≥45	≥45 (unless end-course result)
**CaNa_2_EDTA available**	No	No	No	5 d course (encephalopathic children)	Same as 4
**Duration of DMSA course**	28 d	19 d	19 d	19 d	19 d or 5 d
**Dosing**	28 d BD	7 d TDS + (12 d BD or 12 d TDS)	Same as 2	Same as 2	5 d TDS + (14 d BD or 14 d TDS or end)
**VBLL tests used for differential dosing**	NA—no VBLL-based dosing	Day-7 VBLL (at threshold ≥ 65, continue TDS)	Day-0 VBLL (at threshold ≥ 120, 19-d TDS) (day-7 VBLL test eliminated)	Same as 3	Day-0 VBLL (threshold ≥ 120); course ≥2, also Day-0 VBLL (threshold <65 for 5-d course)
**Frequency of DOT (non-DOT doses administered by caretaker)**	All inpatient, full DOT	Inpatient until late July 2010, then daily outpatient DOT (one dose observed)	Daily outpatient DOT, transition to alternate-day outpatient DOT in November 2010	Alternate-day outpatient DOT	Same as 4
**Protocol at end of a course**					
Immediate retreatment	No immediate retreatment	End-course VBLL ≥ 65	Same as 2	Same as 2	End-course VBLL ≥ 80
Re-test in 2 wk	All	End-course VBLL <65	Same as 2	Same as 2	End-course VBLL ≥ 65 and <80
Re-test in 4 wk	NA	NA	NA	NA	End-course VBLL ≥ 45 and <65
**Protocol with pre-treatment screen or post-chelation follow-up VBLL test**					
Re-test in 2 wk	VBLL ≥ 25 and <45	Same as 1	Same as 1	NA	NA
Re-test in 4 wk	VBLL ≥ 20 and <25	Same as 1	Same as 1	VBLL ≥ 35 and <45	VBLL ≥ 30 and <45
Re-test in 2 mo	VBLL ≥ 15 and <20	Same as 1	Same as 1	VBLL ≥ 20 and <35	VBLL ≥ 20 and <30
Re-test in 3 mo	VBLL ≥ 10 and <15	Same as 1	Same as 1	VBLL ≥ 10 and <20	NA
Re-test in 6 mo	NA	NA	NA	NA	VBLL ≥ 10 and <20
Discharge	VBLL <10	Same as 1	Same as 1	Same as 1	Same as 1

*Flow-chart of protocol 5 provided in Figures S1–S3. VBLL units are micrograms per decilitre.

NA, not applicable.

**Box 1. Process for Zamfara DMSA Protocol Revision**

(1) An expert advisory group* was convened by MSF, comprising non-MSF medical toxicologists and public health professionals with previous experience managing lead poisoning, to guide MSF in building the chelation programme.

(2) As the expert group was convened, MSF started emergency chelation with 28-d BD dosing as per Kosovo and Boston precedents [2].

(3) After the evidence base was reviewed by the expert group, early adjustments to the protocol were made to accord with dominant international practice (treatment duration reduced from 28 to 19 d).

(4) Further adjustments were made based on the following:

- Feedback that was encouraged from programme staff and patients (e.g., patients finding frequency of tests or visits unacceptable, raising compliance challenges).
- Regular review by programme medical staff for possible areas for streamlining.
- Alterations requested by programme team.
- Review of Zamfara data by senior epidemiologist (e.g., to determine whether lengthening chelation-free periods was likely to put children at risk of VBLLs rising to levels that might cause encephalopathy).

(5) Proposals for revision were prepared by the programme advisor, consultant toxicologist, and epidemiologist for the expert group including risk analysis and supporting data.

(6) Proposals were discussed with expert group by teleconference and email to achieve consensus based on literature review and expert opinion.

(7) Agreed revision implemented.

(8) Impact of revision monitored by field team and headquarters support team.

*The expert advisory group comprised the non-MSF-affiliated authors of this paper.

**Box 2. Key Adaptations of Zamfara DMSA Protocol^†^**

**July 2010**

- Length of course reduced from 28 to 19 d to accord with dominant international practice [25].
- Daily dose: 20 mg/kg/d increased to 30 mg/kg/d with review on day 7 of course. Patients continued on 30 mg/kg/d if day 7 VBLL ≥ 65 µg/dl, otherwise decreased to 20 mg/kg/d till end of course.
- Outpatient daily DOT started via outreach clinics for those from remediated villages.
- Next course started immediately if end-course VBLL ≥ 65 µg/dl (previously 2-wk remobilisation interval with any end-course VBLL).

**October 2010**

- Stopped day 7 VBLL and biochemical tests based on ability to predict outcome at end of treatment from initial VBLL, lack of toxicity concerns (ALT and creatinine), and potential to improve community acceptance.

**November 2010**

- IV CaNa_2_EDTA introduced for chelation of encephalopathic children.

- Moved to alternate-day DOT in outpatient clinics.

**March 2011**

- Lengthened chelation-free period dependent on VBLL at end of course.
- 5-d courses of 30 mg/kg/d introduced for those with VBLL of 45–64 µg/dl [24].
- 19-d courses of 30 mg/kg/d for 5 d, followed by 30 mg/kg/d if a first-ever course or if day 0 VBLL ≥120 µg/dl, otherwise 20 mg/kg/d, for a further 14 d.

Note: 20 mg/kg/d and 30 mg/kg/d were both approximate and were actually rounded to 100 mg or 200 mg per dose depending on weight.

^†^ For details of protocol see Table 2.

In hospital, all doses were directly observed (directly observed therapy [DOT]); thus, inpatient location is a proxy for full medication adherence and limited opportunity for further lead exposure. Village environmental remediation commenced concurrent with the first inpatient courses, so for later courses patients were hospitalised only if their village was yet to be remediated, or if they were severely ill. Once a village's environmental lead remediation was completed, non-encephalopathic children were treated as outpatients, initially with one of the two or three daily doses of DMSA directly observed during a clinic visit. Several months later, in response to a growing caseload and the request of parents, only one dose on alternate days was directly observed at the clinic (Table 2); other doses were administered by parents or guardians in the home. The reduced frequency of DOT therefore applied to all children on outpatient treatment and was due to the number of days per week that the clinic operated, not to VBLL or clinical status.

### Inclusions

**Figure 2:**
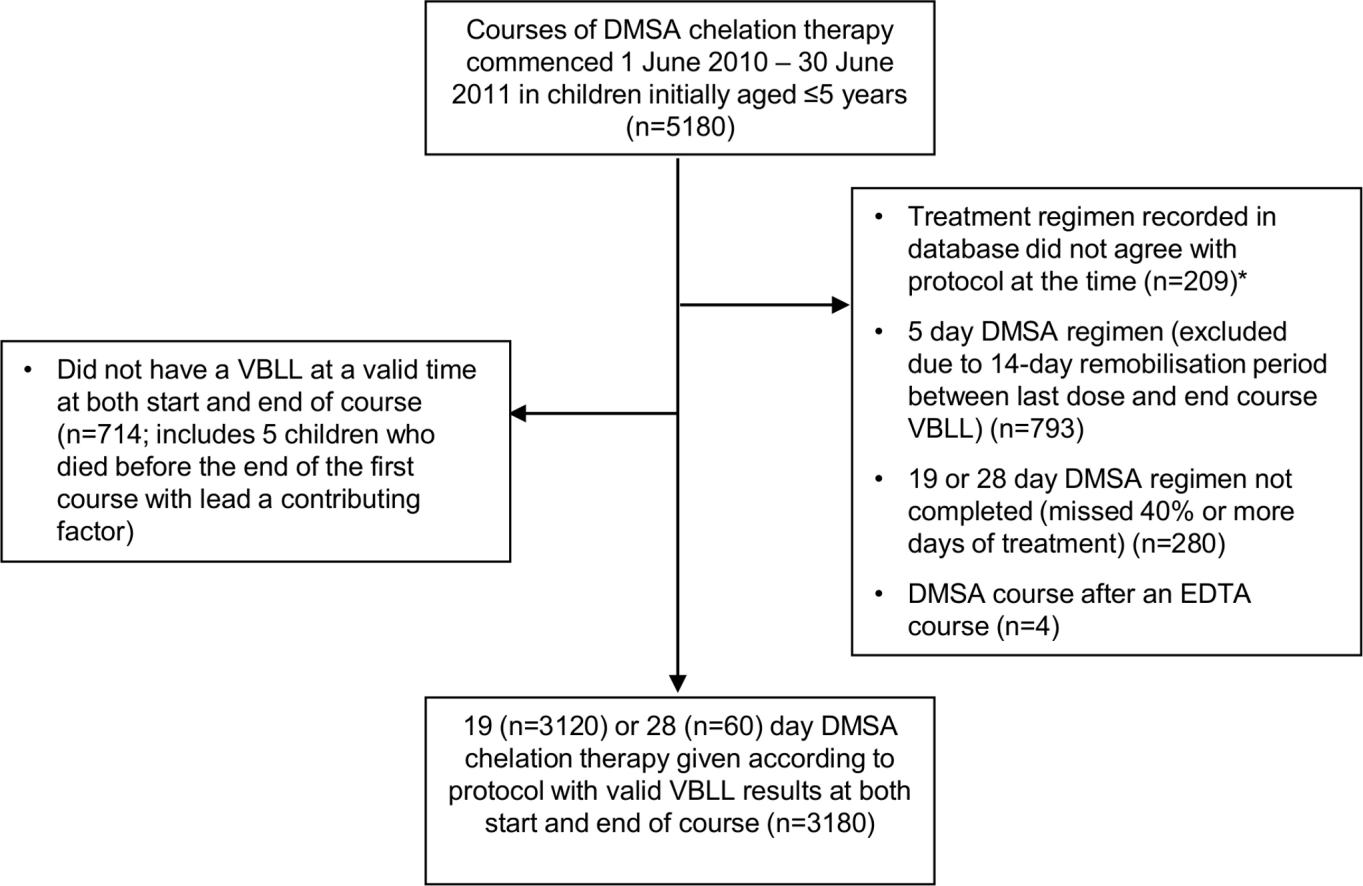
Flow chart of inclusion and exclusion of courses in analysis. *Protocol changeover dates given in Table 2 were strictly applied in reviewing the data, even though implementation in the seven village clinics probably did not occur instantly. Accordingly, some data exclusions were for courses commenced within a few days of a protocol changeover. 92% of exclusions for this reason would have been excluded because of one or more of the other exclusion criteria, which were applied in sequence. EDTA, CaNa_2_EDTA.

All children aged ≤5 y from contaminated villages where remediation was taking place were offered screening. Although all residents of the contaminated villages were considered to have elevated lead exposure, personnel and financial constraints resulted in the chelation protocol being targeted to children ≤5 y of age, the only subgroup in which serious morbidity and mortality had been identified. For statistical analysis, only completed courses of DMSA of 19 or 28 d started between 1 June 2010 and 30 June 2011 with valid pre- and end-course VBLLs recorded (defined below) were included (Figure 2). Except for analysis of overall VBLL decrease per child over the entire period, 5-d DMSA courses (Table 2) were excluded. Included chelation courses were administered to 1,156 children, 856 (74%) of whom were included in a report on association of VBLL with neurological features in 972 children [16].

### Testing

Venepuncture was performed after cleaning the skin with soapy water to minimise lead dust contamination. VBLL was measured using the LeadCare II (Magellan) point-of-care analyser according to manufacturer protocols. For samples with lead levels >65 µg/dl, the upper quantification limit of the device, a blood dilution method utilising donor blood with lead concentration <3.0 µg/dl [41] was developed and implemented on 20 July 2010. Exclusion of courses with VBLL tests conducted before the dilution protocol was finalised did not substantially alter the results of regression modelling; thus, blood samples obtained at all points in the study period were retained. Monthly quality control was provided by the CDC using inductively coupled plasma mass spectrometry (ICPMS): point-of-care values of 120 clinical samples (including 15 samples requiring dilution in the field laboratory) were on average 4.0 µg/dl lower than ICPMS values (limits of agreement [42] [two standard deviations] −19.7 to +11.7 µg/dl).

Haemoglobin was measured with a HemoCue Hb 301 point-of-care testing system prior to 22 November 2010 and with a Sysmex KX-21N Automated Hematology Analyzer from this date. Creatinine and alanine transaminase (ALT) were measured on a HumaLyzer 2000 (Human Gesellschaft für Biochemica und Diagnostica). White blood cells (manual count prior to 22 November 2010, Sysmex KX-21N Automated Hematology Analyzer thereafter) and neutrophils (from 22 November 2010, Sysmex KX-21N Automated Hematology Analyzer) were also measured (white blood cell count was used to calculate absolute neutrophil count from percent), although less consistently because of operational constraints. A sensitivity analysis including a pre-/post-change variable in the main regression model determined that regression analyses were unchanged by the switch in analytical method for haemoglobin. Clinical data were routinely entered into an electronic database specifically designed to support patient care and programme management through automated lists of protocol-dictated actions, test result alerts, and detailed reports (Microsoft Excel 2007).

Adverse events were systematically monitored in that full blood count, creatinine, and ALT were checked regularly at the beginning and end of each course of chelation. Analysis included the completed courses of chelation in the study database. Anecdotal severe adverse events are reported here; minor clinical events (e.g., diarrhoea) were not recorded.

### Definitions

A complete course of DMSA was defined as >60% of days of doses being dispensed. “Pre-chelation VBLL” means first-ever VBLL (chelation naïve). “Pre-course VBLL” (“day 0”) means a VBLL obtained up to 14 d before the course commenced, used to dictate the length and dosing for an individual course. The “end-course VBLL” was obtained from ≤2 d before to ≤10 d after the end of treatment. Age at time of VBLL was categorised as 0 to <6 mo, 6 to <12 mo, 1 to <2 y, 2 to <3 y, and 3 to 5 y [43].

Haematological and biochemical parameters were categorised as follows: haemoglobin as low (<100 g/l if <2 y old; <110 g/l if 2–5 y), high (>130 g/l if <2 y;>140 g/l if 2–5 y), or normal; ALT as normal (0–42 U/l), mildly elevated (42.1–100 U/l), moderately elevated (100.1–1,000 U/l), or severely elevated (>1,000 U/l); creatinine as low (<44 µmol/l), high (>97 µmol/l), or normal; neutrophil count as normal (>1.5×10^9^/l) or as mild (1.0–1.5×10^9^/l), moderate (0.5–0.99×10^9^/l), or severe neutropenia (<0.5×10^9^/l) [44].

“Rebound” refers to the rise in VBLL that may occur in the days to weeks after a chelation-related reduction in VBLL. Rebound typically reflects the internal movement of lead stored in the bone and soft tissue compartments back into the blood following a chelation episode. However, re-exposure to external sources of lead after cessation of chelation may also influence the magnitude of rebound that is recorded.

“DMSA dose regimen” refers to the number of doses per day and number of days of each regimen, while “DMSA administration method” refers to whether the dose was administered in hospital or in the outpatient clinic/at home, and the frequency of direct observation.

### Analysis

We report the association between DMSA chelation and VBLL; clinical outcomes will be reported separately. The primary outcome variable was in line with that used in other studies [24],[25],[26],[27], defined here as end-course VBLL as a percentage of pre-course VBLL (ECP):

ECP* = *(end-course VBLL/pre-course VBLL) × 100% (1)

Thus, an ECP <100% indicates a net decline in VBLL; ECP > 100% indicates that VBLL increased. Since a key goal of chelation treatment is to reduce VBLL, a lower value of ECP is desirable. Geometric means of ECP values for the relevant subset of courses were calculated by fitting a mixed model containing only a constant term using nested random effects (village, patient) to account for repeated measurements in the same individual over multiple treatment courses. The natural logarithm of ECP was the dependant variable, and the exponential of coefficients and the 95% CIs were calculated.

Rebound was quantified as the percentage change between the end-course and subsequent VBLL where there was no administration of DMSA in the interim.

For clinical correlation, as a secondary outcome, absolute increase or decrease of end-course VBLL compared to pre-course VBLL was calculated, with arithmetic means and 95% CIs reported since these data were normally distributed, nested by village and patient.

We used mixed models using nested random effects (village, patient) [45] to assess the influence of factors on ECP and rebound, adjusting as relevant for the following: gender, age at start of each course (categorised), pre-course VBLL (for ECP), end-course VBLL (for rebound), timing of the end-course VBLL measurement relative to first DMSA dose, DMSA dose regimen, whether it was the first course of DMSA, DMSA administration method, and start-of-course haemoglobin.

In analysing the associations between treatment with a course of DMSA and ECP, it was considered important to account for variations in DMSA administration method and dose regimen. These factors were not independent, as protocols and implementation practicalities for both changed over time as the intervention progressed. In addition, the duration of TDS dosing as opposed to BD dosing was based on pre-course VBLL (Table 2), so pre-course VBLL would confound assessment of the role of dose regimen if both were included as independent variables in the model of ECP. Therefore, the primary multivariable model of interest using ECP as the outcome variable was based on 2,262 chelation courses that featured the most prevalent chelation dose regimen: 19 d of treatment (7 d TDS + 12 d BD). A secondary model assessed variations in dose regimen using (coincidentally also) 2,262 courses administered by alternate-day DOT. A subanalysis of courses commenced at VBLL ≥ 120 µg/dl on the 19-d TDS regimen assessed the impact of DMSA at very high VBLLs. Because changes in the protocol with respect to dose regimen and dose administration method were highly correlated with calendar date, the date of a course of chelation was not included as an independent variable in the models. No allowance for multiple testing was performed.

For categorical variables the largest group was used as the base reference, except for haemoglobin, where “normal” was used as the reference. Variables were added to the multivariable model if they were significant in univariate regression at *p* <0.1 or if they were considered clinically or programmatically important. Final multivariable models were selected using backward elimination. *p*-Values were calculated for the strength of association of each variable with the outcome using Wald tests. The DMSA administration method categorical variable was assessed as continuous (in order of decreasing frequency of DOT as shown in the tables) to test for trend. Interaction was assessed where plausible and when sufficient data were available in subgroups to yield precise estimates. All observations were included in the mixed regression models; courses with missing data (*n = *23 [1%], primarily haemoglobin missing) were dropped by the statistical software as not being able to contribute to the model estimation. To estimate how well the variation in the data was explained, we fitted a linear regression, approximating the mixed model for 19-d single-regimen courses, by including village as a variable and adjusting for clustering by patient by using robust standard errors. Standard regression diagnostics were performed using analysis of residuals. Data were analysed using STATA 10.1 (StataCorp).

## Results

**Figure 3:**
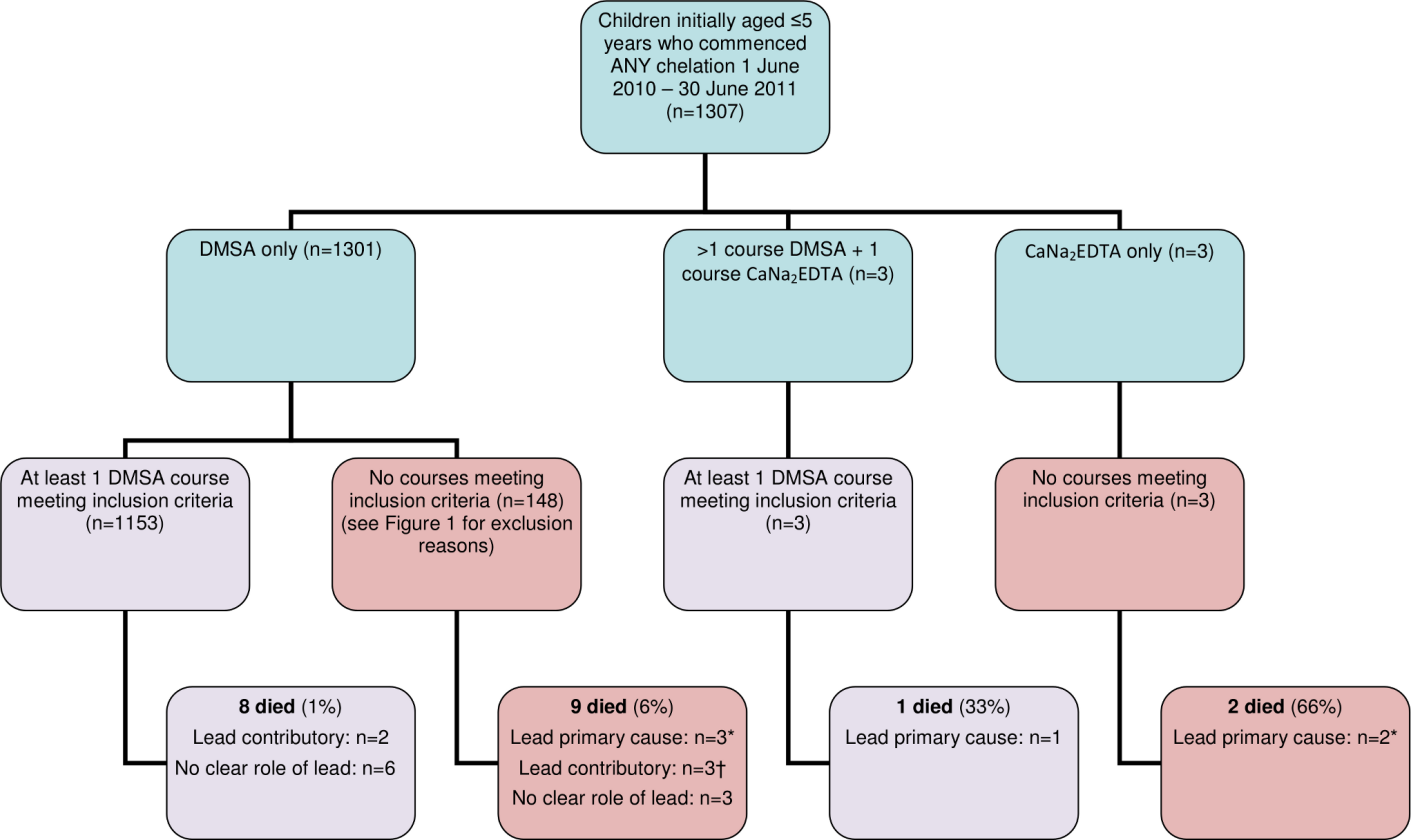
Flow chart of children commencing chelation in period analysed, with inclusion and exclusion in analysis and death outcomes. Lead was attributed as the primary cause of death where there was a high (>100 µg/dl) VBLL within 10 d before death, where there was no other obvious cause, and where lead toxicity could not be excluded as a cause. Lead was attributed as a contributory cause where a serious comorbidity was present (measles, bronchopneumonia, malaria, septicaemia, or severe malnutrition) but with a recent VBLL> 90 µg/dl. Deaths were categorized as “no clear role of lead” where there was another obvious cause (e.g., fell into an open well, anaemic heart failure, or measles) and no recent VBLL> 65 µg/dl. Reasons for not including in the study cohort were not finishing the chelation course (through defaulting or death before end of course) or no VBLL recorded at end of course. *All died during first treatment course. †Two died during first treatment course.

A total of 3,180 DMSA treatment courses of 19 or 28 d commenced between 1 June 2010 and 30 June 2011 were included (Figure 2). Included courses were administered to 1,156 children, nine of whom had died by end of June 2011. One death was likely solely due to lead encephalopathy; eight children died from other primary causes, but two of these children had had lead levels> 90 µg/dl within 10 d of death (Figure 3). Amongst children excluded from the primary analysis, five additional deaths occurred during the first chelation course with likely primary cause of death listed as lead toxicity (most recent VBLL 216–460 µg/dl), including two commenced on CaNa_2_EDTA. Another three children died because of another primary cause but had had lead levels> 90 µg/dl within 10 d of death. These eight are not included in the treatment analysis as they did not have an end-course VBLL or did not meet other inclusion criteria (Figure 3). First treatment courses (882 courses) constituted 28% of those analysed; the first administered course did not meet the inclusion criteria for 274 children for whom later courses could be included. 66% of children had more than one course, with some receiving up to 15 treatment courses of any length (median 3 courses, interquartile range 1–4) during the 13 mo analysed. The male:female ratio for the number of courses started was 1.06. More than half of the patients were aged 3–5 y (Table 3). Pre-course VBLLs ranged from 45 to 345 µg/dl (median 60, interquartile range 52–92). 36% and 6% of courses were commenced with a VBLL ≥80 µg/dl and ≥120 µg/dl, respectively.

**Table 3:** Baseline characteristics.

Characteristic	Pre-Chelation† (*n = *1,156 Children)	Pre-Course (*n = *3,180 Courses)
**Age at start of course**		
0 to <6 mo	48 (4%)	63 (2%)
≥6 mo to <1 y	128 (11%)	300 (9%)
≥1 y to <2 y	190 (16%)	689 (22%)
≥2 y to <3 y	115 (10%)	342 (11%)
≥3 y to ≤5 y	675 (58%)	1,786 (56%)
**Sex**		
Male	599 (52%)	1,638 (52%)
Female	557 (48%)	1,542 (48%)
**VBLL (μg/dl)**		
45–64.9	709 (61%)	1,856 (58%)
65–79.9	68 (6%)	187 (6%)
80–119.9	295 (26%)	945 (30%)
120–199.9	69 (6%)	174 (5%)
200–345	15 (1%)	18 (1%)

Data are *n* (percent).

† First course with full data for each child, not all first-ever course.

Of 19- or 28-d courses completed between June 2010 and end June 2011, only 82% had both pre-course and end-course VBLLs that were classified as valid measures for that treatment event point. This was due to the logistical challenges of reaching villages (e.g., during the rainy season, resulting in delayed blood tests), lack of stock of laboratory test supplies due to high demand, and, in the early months, challenges with maintaining adequately low ambient temperature for laboratory equipment in the harsh environment. Excluded courses without an end-course VBLL had a slightly (2.4 µg/dl) but significantly (rank-sum *p = *0.008) higher pre-course VBLL than those with both tests recorded.

### ECP after a DMSA course: Overview

**Figure 4:**
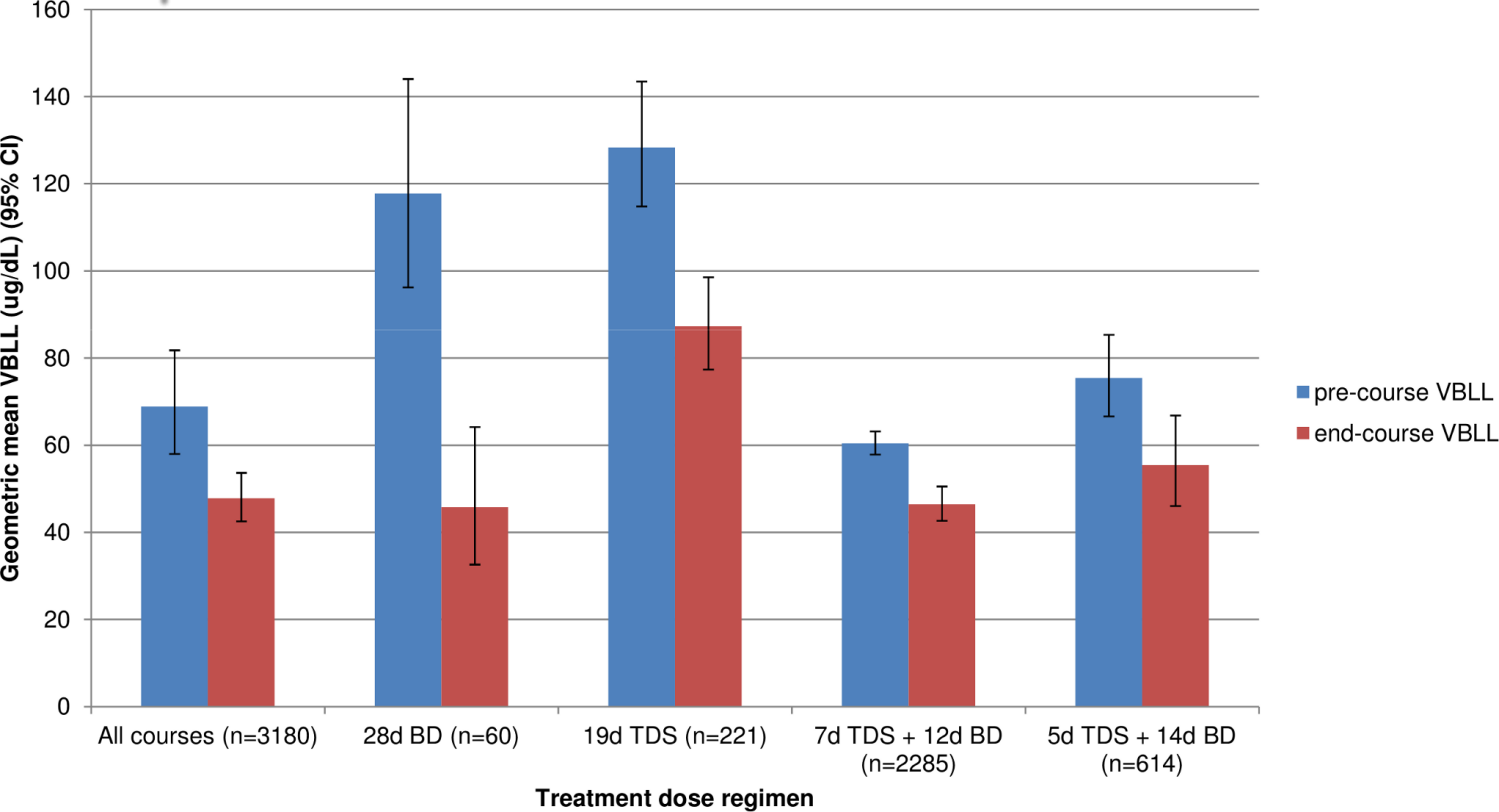
Geometric mean (95% CI) of absolute pre-course and end-course VBLL for all courses nested by village and patient, by treatment dose regimen.

**Figure 5:**
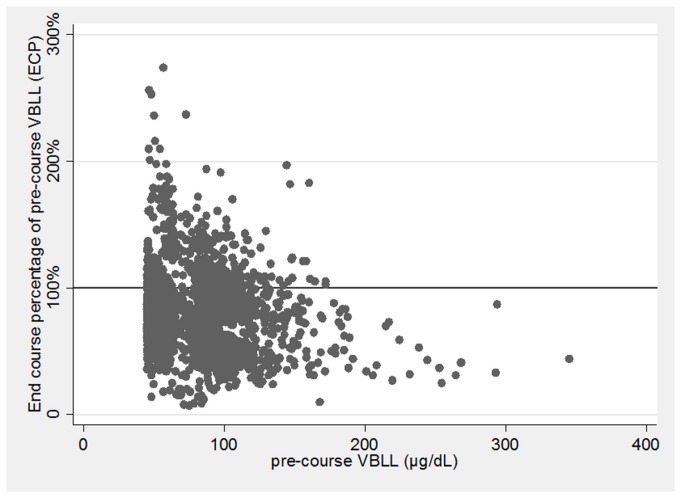
Unadjusted end-course VBLL as a percentage of pre-course VBLL (ECP) by pre-course VBLL (μg/dl) (*n = *3,180).

**Figure 6:**
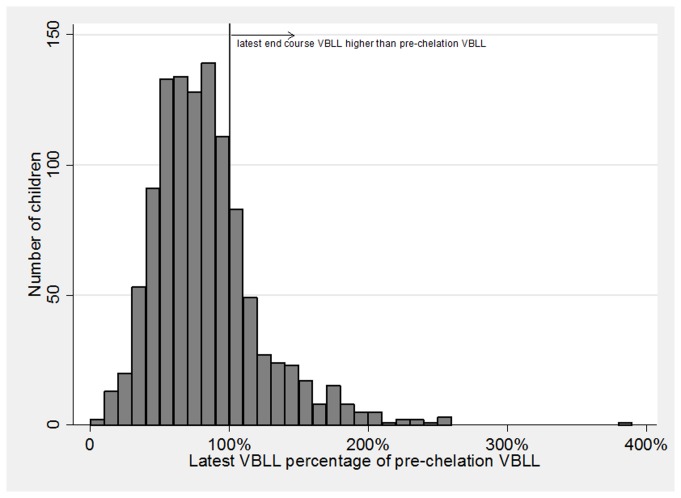
Histogram of latest VBLL during the entire study period as a percentage of pre-chelation VBLL per child. As the role of repeated courses on combined overall change cannot be extracted, change includes effect of all courses (e.g., 5-d and CaNa_2_EDTA courses). This does not represent the end of treatment in most cases, but rather an interim measure at the end of the study period.

For all 3,180 courses (Table 4), ECP had a geometric mean of 74.5% (95% CI 69.7%–79.7%) (Table 5), with a mean absolute decrease of 22.3 µg/dl (95% CI 8.1–36.5) (Figure 4). At the end of a single treatment course, ECP varied from 7% to 274%. Instances in which ECP exceeded 100% occurred predominantly when the pre-course VBLL was less than 120 µg/dl (Figure 5). At the end of their most recent course, 71% of children had a VBLL lower than their pre-chelation VBLL; for most children this was an interim measure at the end of the analysis period but not the end of their treatment intervention (Figure 6).

**Table 4:** Number of courses by dose regimen and administration regimen.

Regimen	>95% Course in Hospital	50%–95% Course in Hospital, Then Outpatient	≥80% Course Outpatient Daily DOT	Course via Outpatient Mixed DOT (Daily then Alternate Day)	≥80% Course Outpatient Alternate-Day DOT	Total (Percent of Total)
28 d BD	60 (100%)	0	0	0	0	60 (2%)
19 d TDS	27 (12%)	9 (4%)	86 (39%)	1 (0%)	98 (44%)	221 (7%)
7 d TDS + 12 d BD†	72 (3%)	89 (4%)	414 (18%)	137 (6%)	1,573 (69%)	2,285 (72%)
5 d TDS + 14 d BD	0	0	0	0	614 (100%)	614 (19%)
Total	159 (5%)	98 (3%)	500 (16%)	138 (4%)	2,285 (72%)	3,180

Data are *n* (percent of regimen). Categories differentiate full DOT (here meaning entire course was taken in hospital) compared with various combinations of partial DOT when a child was discharged mid-course to receive the remainder of the course in the community, either with daily or alternate-day DOT of one of the day's doses and the remainder administered by the caretaker at home.

† Courses in this row included in model in Table 6.

**Table 5:** Association of dose regimen and dose administration method with geometric mean ECP (expressed as percent).

Regimen	>95% Course in Hospital (*n = *159)	50%–95% Course in Hospital, then DOT (*n = *98)	≥80% Course via Outpatient Daily DOT (*n = *500)	Course via Outpatient Mixed DOT (Daily then Alternate Day) (*n = *138)	≥80% Course via Outpatient Alternate-Day DOT (*n = *2,285)	Total (Percent of Total) (*n = *3,180)
28 d BD (*n = *60)	38.8% (32.4%–46.4%)	—	—	—	—	38.8% (32.4%–46.4%)
19 d TDS (*n = *221)	55.5% (46.2%–66.5%)	81.3% (57.7%–114.6%)	78.4% (73.2%–83.9%)	95%	72.0% (66.3%–78.2%)	73.1% (69.5%–76.9%)
7 d TDS + 12 d BD (*n = *2,285)	49.6% (39.5%–62.2%)	55.7% (37.6%–82.5%)	76.0% (63.3%–91.3%)	75.0% (71.8%–78.3%)	83.9% (79.6%–88.4%)	78.3% (73.6%–83.3%)
5 d TDS + 14 d BD (*n = *614)	—	—	—	—	74.6% (67.4%–82.6%)	74.6% (67.4%–82.6%)
Total (*n = *3,180)	47.7% (39.7%–57.3%)	61.6% (43.9%–86.3%)	76.4% (64.4%–90.7%)	75.1% (71.9%–78.4%)	79.3% (73.8%–85.2%)	74.5% (69.7%–79.7%)

Data are ECP (95% CI). See Table 4 for number of courses per cell.

For 882 first-ever treatment courses that met inclusion criteria, the geometric mean ECP was 68.0% (95% CI 59.2%–78.2%), with a mean absolute decrease of 29.4 µg/dl (95% CI 8.8–50.0). For 2,298 subsequent (after-first) courses, the mean ECP was 80.2% (95% CI 77.3%–83.3%), with a mean absolute decrease of 12.5 µg/dl (95% CI 10.5–14.5).

For all 19-d courses (*n = *3,120) mean ECP was 75.2% (95% CI 70.5%–80.3%), with a mean absolute decrease of 22.2 µg/dl (95% CI 7.1–37.3). The lowest mean ECP (38.8% [95% CI 32.4%–46.4%]) was achieved in the 60 courses of 28-d duration administered entirely on an inpatient basis. All outpatient administration methods (fewer doses DOT) had a higher mean ECP. The mean ECP for inpatients with all doses DOT was 47.7% (95% CI 39.7%–57.3%) compared with 79.3% (95% CI 3.8%–85.2%) for outpatient courses given as alternate-day DOT (Table 5).

### 19-d Single-Regimen Courses

For 19-d courses using the 7 d TDS + 12 d BD regimen (*n = *2,262) (Table 6), the final adjusted model showed ECP was significantly associated with age, pre-course VBLL, interval since previous course, administration method, timing of end-course VBLL measurement, and haemoglobin. After adjustment for the other variables included in the final model, first treatment courses were not associated with any difference in ECP compared with subsequent courses. Gender was not associated with change in ECP but was retained in the final model as a key demographic factor. ECP was 12%–17% higher in children aged <3 y than in those aged 3–5 y (Table 6). Pre-course VBLL was strongly associated with ECP: 0.54% lower ECP for every 1 µg/dl increase in pre-course VBLL within the range 45 to <120 µg/dl, with geometric mean pre-course VBLL 60.5 µg/dl (95% CI 57.2–63.2). For each extra chelation-free day since the end of the previous course, there was a 0.05% larger decrease in ECP. ECP was lowest if VBLL was measured immediately at the end of a treatment course compared to a few days after a course ended. Low haemoglobin at the start of a course was associated with a relative increase (6.7%) in ECP compared to normal haemoglobin. Linear regression including village and clustered by patient indicated that the model explained approximately 26% (*R* ^2^ *= *0.255) of the variation in the data. Standard regression diagnostics indicated that residuals were normally distributed. There was no evidence of interaction between pre-course VBLL and administration method (*p = *0.11). Administration methods that offered greater opportunity for DOT by clinic staff were associated with larger reductions in ECP during a course (test of trend *p* <0.001).

**Table 6:** Mixed model of ECP using nested random effects for a 19-d (7 d TDS + 12 d BD) DMSA chelation course (*n = *2,285 in unadjusted analysis; *n = *2,262 in adjusted analysis limited to patients with data on all covariates).

Characteristic	*N* *	Unadjusted Estimate (95% CI)	Adjusted Estimate† (95% CI)	*p*-Value (Adjusted Model)
**Age at start of each course**				<0.001
0 to <6 mo	38	3.7% (−4.4%, +11.8%)	12.4% (+4.8%, +19.9%)	
6 to <12 mo	211	10.6% (+6.9%, +14.4%)	14.7% (+10.9%, +18.4%)	
1 to <2 y	398	11.7% (+8.7%, +14.7%)	17.1% (+14.0%, +20.3%)	
2 to <3 y	226	10.4% (+6.6%, +14.1%)	11.8% (+7.9%, +15.6%)	
3 to ≤5 y	1,412	Referent	Referent	
**Sex**				0.44
Male	1,162	Referent	Referent	
Female	1,123	−0.70% (−3.0%, +1.6%)	−0.90% (−3.2%, +1.4%)	
**Course number**				0.14
First	540	−8.6% (−11.0%, −6.1%)	−2.1% (−5.0%, +0.7%)	
Second or later	1,745	Referent	Referent	
**Pre-course VBLL (per 1 μg/dl)**	2,285	−0.65% (−0.70%, −0.60%)	−0.54% (−0.60%, −0.49%)	<0.001
**Days since last course ended (per day)**	2,285	0.07% (+0.03%, +0.12%)	−0.05% (−0.10%, −0.01%)	0.017
**Administration method**				<0.001
>95% course in hospital	72	−31.9% (−37.6%, −26.3%)	−15.2% (−20.8%, −9.6%)	
50%–95% of course in hospital, then outpatient	89	−21.8% (−26.9%, −16.6%)	−12.1% (−17.2%, −7.1%)	
≥80% course via outpatient daily DOT	414	−15.0% (−17.8%, −12.3%)	−8.4% (−10.9%, −5.8%)	
Course via outpatient mixed DOT (daily then alternate day)	137	−9.7% (−13.9%, −5.5%)	−8.2% (−12.2%, −4.2%)	
≥80% course via outpatient alternate-day DOT	1,573	Referent	Referent	
**Timing of end-course test, relative to start‡**				0.004
Day 17–19	1,614	Referent	Referent	
Day 20–26	645	4.4% (+2.1%, +6.7%)	3.2% (+1.2%, +5.2%)	
Day 27–29	24	9.7% (−0.4%, +19.8%)	6.7% (−2.0%, +15.4%)	
**Haemoglobin (pre-course)**				<0.001
Normal	895	Referent	Referent	
Low	1,355	6.7% (+4.5%, +8.9%)	6.6% (+4.5%, +8.6%)	
High	14	−10.4% (−23.5%, +2.7%)	−7.9% (−19.1%, +3.4%)	

A positive value of ECP indicates an increase in VBLL; a negative value of ECP indicates a decrease in VBLL. DOT by clinic staff; non-DOT doses administered by caretaker.

*Total patients in unadjusted analyses* = *2,285. Final model (*n = *2,262) excludes those with missing data (1%).

† Adjusted for all other variables shown in the table.

‡ An end-course VBLL was accepted if up to 2 d before and up to 10 d after last dose of DMSA.

### Influence of Dose Regimen during 19-d Courses

The relative ECP for different dose regimens was examined within the subset of courses administered by alternate-day DOT (*n = *2,262). After adjustment for age, first course versus subsequent course, days since previous course ended, timing of end-course VBLL measurement, and haemoglobin (all associations similar to those in Table 6), the 19-d TDS regimen yielded an 18.6% lower ECP—and the 5 d TDS + 14 d BD regimen yielded a 4.4% lower ECP—than the 7 d TDS + 12 d BD regimen. This finding suggests that dosing three times a day for the whole course might yield the greatest reduction in VBLL. However, differences in ECP suggested by TDS versus BD dosing may instead just reflect the influence of pre-course VBLL differences (in light of the role of pre-course VBLL demonstrated in the model in Table 6). The geometric mean pre-course VBLL was 140.9 µg/dl (95% CI 134.8–147.3) for 19-d TDS courses, 58.4 µg/dl (95% CI 58.3–60.4) for 7 d TDS + 12 d BD, and 75.5 µg/dl (95% CI 66.6–85.6) for 5 d TDS + 14 d BD, relative levels that are coherent with the ECP differences.

### Courses Commenced with Very High VBLLs

The geometric mean ECP for 148 courses commenced at VBLL ≥ 120 µg/dl using the 19-d TDS regimen was 67.8% (95% CI 61.7%–74.4%). A mixed model confined to these courses commenced with high pre-course VBLL showed that after adjustment for age and haemoglobin (associations consistent with main model), three factors were strongly associated with ECP. A first course was associated with lower ECP than a subsequent course (−21.8%, 95% CI −34.1% to −9.5%), a finding not seen in the larger models that included initial VBLL values from 45 µg/dl. An increase in the interval between chelation courses was associated with a 0.7% per day (95% CI −1.1% to −0.3%) decrease in ECP. Every 1 µg/dl increase in pre-course VBLL in this subgroup was associated with a 0.20% (95% CI −0.33% to −0.07%) decrease in ECP.

The geometric mean ECP for 159 almost exclusively inpatient courses of 19 or 28 d (>95% of course in hospital) was 47.7% (95% CI 39.7%–57.3%) (Table 5), with a mean absolute decrease of 56.2 µg/dl (95% CI 40.7–71.6). For 145 inpatient-only courses that were also first-ever courses the decline was slightly greater, yielding an ECP of 44.9% (95% CI 35.4%–56.8%). Modelling indicated that the influence of pre-course VBLL on ECP was attenuated in the subset of patients undergoing a first course of DMSA administered entirely on an inpatient basis. In this subset (*n = *145, geometric mean pre-course VBLL 106.2 µg/dl, 95% CI 87.1–129.4), after adjustment for age, for every 1 μg/dl increase in pre-course VBLL there was only a 0.09% decrease in ECP (95% CI 0.02% to 0.16%; *p = *0.014).

### VBLL Rebound

**Figure 7:**
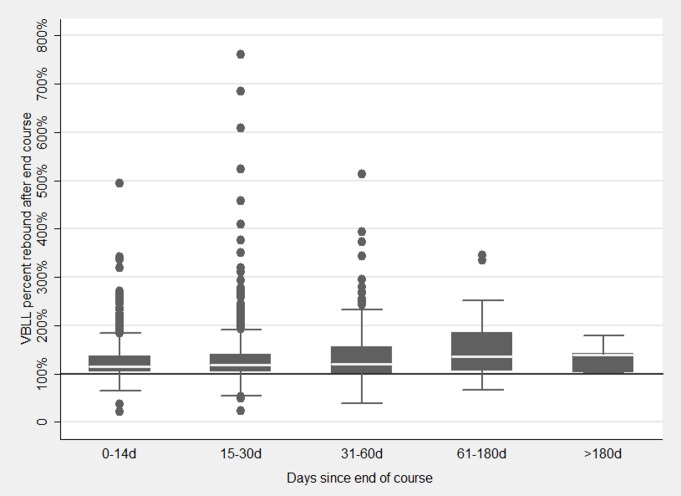
VBLL rebound by the number of days between end-course VBLL and next test. VBLL rebound is VBLL at the next test after the end of a 19-d course and a chelation-free period, as a percentage of end-course VBLL.

In 2,444 pairs of tests that included a test at the end of a 19-d course of any dose regimen and the subsequent test following an interval without chelation (median 19 d, range 1–262), the geometric mean (nested by village and patient) of the VBLL rebound was 122.5% (95% CI 118.9%–126.3%), with 82% of tests showing VBLL rebound above the end-course VBLL (Figure 7). Factors significantly associated with higher rebound included higher VBLL at the start and end of the course just taken, and more chelation-free days between courses and tests. The largest percentage change was with the VBLL at the end of the course: the lower the absolute value of the end-course VBLL, the lower the percent rebound (Table 7). Exclusion of very high rebound outliers did not attenuate the strong associations in the model.

**Table 7:** Mixed model of VBLL rebound following a course of chelation (nested random effects; *n = *2,444).

Course Characteristic	Unadjusted Estimate (95% CI)	Adjusted Estimate (95% CI)	*p*-Value
VBLL at start of course (per 1 µg/dl)	0.34% (+0.26%, +0.43%)	0.73% (+0.65%, +0.81%)	<0.001
VBLL at end of course (per 1 µg/dl)	−1.41% (−1.56%, −1.27%)	−1.95% (−2.10%, −1.80%)	<0.001
Interval since prior chelation course (per day)	−0.20% (−0.28%, −0.13%)	−0.15% (−0.21%, −0.08%)	<0.001
Days from when this course ended to when the “next” VBLL was measured (per day)	0.23% (+0.13%, +0.33%)	0.22% (+0.13%, +0.30%)	<0.001
Constant coefficient		167.06 (157.08, 177.04)	<0.001

VBLL rebound is VBLL at the next test after the end of a 19-d course as a percentage of end-course VBLL.

### Adverse Events

There were no anecdotal reports of severe clinical adverse events related to DMSA. One child developed pneumonia while receiving DMSA via nasogastric tube. This was diagnosed clinically on the basis of fever, hypoxia, and unilateral chest crepitations on auscultation as there were no radiology services available. The child had an AVPU (alert, voice, pain, unresponsive) [46] score of U, and the pneumonia may have been secondary to aspiration in a deeply unconscious child without sufficient gag reflex, though this was not confirmed. Alternatively, it may have represented a non-aspiration nosocomial pneumonia in a critically ill child. The child was treated with oxygen, IV ampicillin, and metronidazole and recovered. Three deaths in children with an AVPU score of P or U receiving DMSA by nasogastric tube (11% of 26 children) occurred in the first few days of treatment with severe lead poisoning as the primary cause; these patients were not included in the analysed courses as there was no end-course VBLL (Figure 3).

The incidence of severe neutropenia (neutrophil count <0.5 × 10^9^/l) after any course of DMSA was low (0.4%) and was comparable to that seen before commencement of DMSA (0% severe neutropenia pre-chelation, 0.3% severe neutropenia before any individual course). Of six children with severe neutropenia at the start of a course, three had normal neutrophil counts and three no result when the end-course VBLL was measured, but nine of the ten children with severe neutropenia at the end of a course did not have a result recorded for the start of that course. There were no recorded cases of severely elevated ALT (>1,000 U/l) either in chelation-naïve patients or after DMSA had been started. Moderately elevated ALT (100.1–1,000 U/l) was present in 1.0% of chelation-naïve children (range 4–256 U/l), increasing slightly to 1.8% after one course of DMSA, and staying constant at 2.4% after multiple courses (ALT range 3.2–498 U/l). Doubling or more of ALT during a course of DMSA occurred in 123 courses (of 2,588 with data, 4.8%). No clinical laboratory result required discontinuation of further chelation treatment in any child.

## Discussion

We report the largest cohort, to our knowledge, of children treated with chelation therapy for severe lead poisoning to date. Our experience demonstrates that oral DMSA may be suitable for use as a single agent in patients with severe lead poisoning, particularly in resource-limited and/or remote settings. The establishment of chelation treatment for lead-poisoned children—in parallel with an environmental remediation programme to reduce ongoing exposure—in villages in rural northern Nigeria was associated with a large and rapid decrease in the number of deaths due to lead poisoning. Only six deaths thought to be solely due to lead poisoning occurred during the 13-mo period analysed, compared with over 400 fatalities in the three preceding months (Figure 3). The primary end point of interest in our analysis was quantitative reduction of VBLL achieved during a course of oral DMSA chelation, expressed as ECP. Lower VBLLs are associated with fewer clinical and sub-clinical features of lead poisoning, and VBLLs ≥80 µg/dl are associated with encephalopathy [16]. A clinically desirable lower value of ECP was more strongly associated with first-ever courses of chelation than with subsequent courses, with inpatient than with outpatient courses, and with a greater degree of DOT. In a multivariable regression analysis of outpatient chelation courses, ECP was lower with older age, higher pre-course VBLL, greater level of DOT, and higher haemoglobin. The magnitude of rebound in VBLL after completion of a course was positively correlated with pre-course VBLL. No serious adverse drug effects attributed to oral DMSA were observed. A moderate increase in ALT that did not require discontinuation of chelation occurred in <1.5% of children.

Paediatric lead encephalopathy not treated with chelation has historically been associated with a case mortality rate of approximately 65% [47]. Use of enhanced supportive care and the parenteral chelating agents CaNa_2_EDTA and dimercaprol reduced this to <5% by the 1960s [48]. In the present cohort, remediation, supportive care, and chelation resulted in an overall all-cause mortality in all children commencing any chelation of 1.5% (20/1,307; Figure 3). The case mortality rate in severe paediatric lead poisoning was <2.5% among 479 children (379 in study cohort; Table 3) who started chelation with initial VBLL ≥ 80 µg/dl. This rate is based on a conservative estimate of 11 (six primary, five possible contributory) lead poisoning deaths in this group (Figure 3). This is a marked decrease from the 48% mortality in probable or suspected child cases documented until mid-May 2010, before the chelation programme was initiated [16].

Across the entire study period, 71% of the children experienced a net reduction in VBLL and 29% a net increase in VBLL after 1–15 courses. These values reflect children at all stages of chelation during the study period, many of whom continue to receive treatment as part of the ongoing medical programme. The overall mean ECP value of 68.0% (corresponding to a decrease in VBLL of 32%) for first-ever courses of DMSA was higher than that observed in other studies of paediatric lead chelation, in which ECP values of 19% to 60% were reported (Table 8) [39],[49],[50],[51]. By comparison, the subset of children who received their first course as inpatients experienced a geometric mean ECP of 44.9%. This latter value, similar to that reported in other studies in resource-rich countries, likely reflects the impact of assured medication compliance in a lead-free environment.

**Table 8:** Paediatric lead chelation: ECP as a function of drug regimen and dose administration method.

Study	Drug	Dose Regimen	Drug Administration Method	Number of Treatment Courses	Mean Pre-Course VBLL (μg/dl)	ECP
Chisolm (1968) [48]	CaNa_2_EDTA (i.m.) + dimercaprol (i.m.)	12.5 mg/kg q 4 h × 72 h and 4 mg/kg q 4 h × 72 h	Inpatient	8	272	19%
Chisolm (1968) [48]	CaNa_2_EDTA (i.m.)	12.5 mg/kg q 4 h × 72 h	Inpatient	7	163	47%
Chisolm (1990) [49]	CaNa_2_EDTA (i.m.)	500 mg/m^2^ BD × 5 d	Inpatient	18	55	60%
Graziano et al. (1992) [26]	CaNa_2_EDTA (IV)	500 mg/m^2^ BD × 5 d	Inpatient	4	54	55%
Graziano et al. (1992) [26]	DMSA (po)	350 mg/m^2^ TDS × 5 d + 350 mg/m^2^ × 15 d	Inpatient (5 d) then outpatient (15 d, no DOT)	6	52	50%
Liebelt et al. (1994) [39]	DMSA (po)	10 mg/kg TDS × 5 d + 20 mg/kg BD × 14 d	Outpatient (no DOT)	7	51	42%
Liebelt et al. (1994) [39]	DMSA (po)	10 mg/kg TDS × 5 d + 20 mg/kg BD × 14 d	Outpatient (no DOT)	23	31	40%
Chisolm (2000) [50]	DMSA (po)	350 mg/m^2^ TDS × 5 d + 350 mg/m^2^ BD × 21–23 d	Inpatient and outpatient	66	37	35%
TLC Trial Group (1998; 2000) [28],[51]	DMSA (po)	350 mg/m^2^ TDS × 7 d + 350 mg/m^2^ BD × 19 d	Outpatient (no DOT)	396	26	57%
Current study	DMSA (po)	10 mg/kg TDS × 19 d; or 10 mg/kg × 7 d + 10 mg/kg BD × 12 d; or 10 mg/kg BD × 28 d	Inpatient (>95% course as inpatient)	159	101	48%
Current study	DMSA (po)	10 mg/kg TDS × 19 d; or 10 mg/kg TDS × 7 d + 10 mg/kg BD × 12 d; or 10 mg/kg TDS × 5 d + 10 mg/kg BD × 14 d	Outpatient (>80% as outpatient) with alternate-day DOT	2,285	62	79%

po, per os (orally); i.m., intramuscular; q, every.

Nearly one-third of the children in this study finished the 13-mo study period with a VBLL that exceeded their initial pre-chelation VBLL. This suggests that despite remediation and community education, outpatient re-exposure to environmental lead contamination was widespread. The implementation of safer mining practices and mitigation of some re-exposure pathways, such as “take-home exposure” from parents engaged in high-risk artisanal mining activities, were challenging for the community. Additional potential pathways of lead re-exposure were not quantified, such as potential contamination of the food supply and the use of lead-contaminated mud bricks for housing. The value of ECP achieved with outpatient courses of chelation, which constituted most of the courses in this study, would likely have been better if all potential sources of re-exposure to lead could have been mitigated.

Our finding of an incremental decrease in ECP as pre-course VBLL increased was unexpected. Pharmacokinetic studies have suggested that the in vivo conversion of DMSA to an active chelating moiety, and its subsequent renal clearance, may be diminished in children with significant lead poisoning, so a higher ECP might be expected in children with higher VBLLs [52],[53]. In a series of 17 adults with pre-course VBLLs between 50 and 140 µg/dl, higher pre-course VBLL was correlated with a higher ECP after 5 d of DMSA treatment [25]. One potential explanation for our findings is that in patients who present with high initial VBLLs, DMSA results in more efficient mobilisation and excretion of labile blood lead pools than in those starting chelation at a lower VBLL. It is also possible that children with higher pre-course VBLLs may have been less active and ambulatory, and therefore sustained less concomitant re-exposure to lead during each chelation course. This effect of possible re-exposure may have persisted even after adjustment for location of treatment (Table 6). As may be seen in Figure 5, some chelation courses initiated with a pre-course VBLL of less than approximately 120 µg/dl were associated with an ECP of >100% (i.e., an end-course VBLL greater than the pre-course VBLL). This finding is not generally observed in the absence of re-exposure.

In patients with VBLL ≥ 120 µg/dl, it was notable that the ECP achieved by the first course was substantially better than that achieved by subsequent courses. This finding has also been observed with lead chelation of severely poisoned patients treated with CaNa_2_EDTA [12],[54],[55]. Similarly, within courses of chelation with CaNa_2_EDTA and DMSA, the largest change in VBLL and urinary lead excretion is usually observed during the first 1–2 d of treatment [26],[27],[55],[56]. Overall, this pattern is consistent with a mobilisation capacity of chelating agents that is most efficient for labile lead pools that are less tightly bound to cellular targets.

For each extra day between 19-d courses there was a 0.05%–0.20% lower ECP at the end of the subsequent course (depending on subset analysis), such that a 30-d gap between courses was estimated to result in an additional decrease in ECP on the subsequent course of approximately 2%–6%. This suggests an increased pharmacodynamic effect of DMSA in mobilising lead as the interval between courses increases. Graziano et al. showed that patients who received no further chelation after a 5-d course of DMSA 1,050 mg/m^2^ per day rebounded to a higher VBLL 1 wk post-course than those who received either 350 mg/m^2^ or 700 mg/m^2^ of DMSA per day over the subsequent 14 d [26]. Further research is needed to determine the optimal chelation-free period between courses, as the clinical impact of a given VBLL without chelation for a longer period should be weighed against potentially achieving a larger subsequent VBLL decrease. There are also potential cost savings if the same long-term impact on VBLL can be achieved with greater spacing between courses without the risk of increased neurological morbidity.

There were only 60 courses of the 28-d regimen included in our analysis, compared with 3,120 19-d courses. The 28-d inpatient courses were given at a very early emergency stage of the programme to some of the most severely poisoned children. Because 19-d course regimens were selected based on pre-course (and initially day 7) VBLL, the influence of pre-course VBLL and various treatment regimens could not be dissected to ascertain whether the apparently greater impact of the 28-d regimen on ECP was independent of pre-course VBLL and other factors. This would be an interesting direct comparison for future study. Thus, while the decreases in ECP were greater with the 28-d regimen, we do not conclude that there is sufficient evidence to support its adoption in place of 19-d regimens.

With the most widely used dose regimen (7 d TDS + 12 d BD), administration of 80% of the course by daily DOT resulted in an ECP of 76.0%; this increased to 83.9% when alternate-day DOT was instituted (see Table 5), possibly because of decreased medication adherence for the doses administered at home by the caretaker. This protocol change was nonetheless judged necessary by the expert advisory group based on the limited capacity of the outpatient clinic in the face of an increasing caseload, and the requests of parents who found it difficult to return to the clinic on a daily basis with their child. The expert advisory group considered that a failure to switch to alternate-day DOT might have resulted in many parents abandoning chelation treatment altogether. An MSF health promotion and community mobilisation team continue to work in Zamfara to support outpatient treatment adherence.

We noted an inverse relationship between haemoglobin concentration and ECP (Table 4), similar to the smaller decrease in VBLL in iron-deficient children described by Ruff et al., although that study used CaNa_2_EDTA as the chelating agent [57]. Anaemia in the setting of lead intoxication is associated with an increased percentage of lead in the plasma fraction of blood [58],[59]. Higher plasma lead may be associated with greater loading of lead into the soft tissues and skeleton, reducing the impact of chelation on VBLL. The adjusted model that included haemoglobin, in addition to age and DMSA dose factors, explained about 26% of the variation in ECP (Table 6), which—not unexpectedly, given the complex setting of the study—indicates that other relevant but unmeasured variables exist.

Our findings indicate that in obtunded children with lead encephalopathy, oral administration of DMSA via nasogastric tube was feasible and associated with a reduction in VBLL. It carries the risk of aspiration, although only one possible case of a complication arising from aspiration was documented among 26 (14 in analysed courses, 12 in excluded courses) courses of DMSA administered by nasogastric tube in this cohort. Three of 18 deaths in children commenced on DMSA occurred in children receiving it by nasogastric tube; these incomplete courses were not included in the ECP analysis. In developed countries, children with severe lead encephalopathy are treated in intensive care units with CaNa_2_EDTA singly or in combination with dimercaprol. The experience in the present cohort suggests that nasogastric administration of DMSA was an acceptable alternative, which is particularly pertinent to resource-limited and/or remote settings. Optimally, depending on the resource setting, an unconscious child would be intubated, diminishing the risk of aspiration from nasogastric administration of DMSA.

The mild rise in ALT observed during DMSA chelation is consistent with other reports [24]. No ALT exceeded 500 U/l, and no overt hepatic dysfunction was observed. Drug-induced neutropenia was not detected, and there was no adverse effect of chelation on creatinine. Adverse clinical symptoms sometimes associated with DMSA therapy in other reports, including gastrointestinal discomfort and mild rash [25], were not systematically documented. Severe adverse drug reactions, such as anaphylaxis or mucocutaneous drug eruptions, did not occur. Overall, the acceptable safety profile of oral DMSA chelation in this study was similar to that reported in other studies of lead chelation.

### Limitations

This is a retrospective analysis of clinical data with the associated limitations. No allowance for multiple testing was performed; false-positive results are possible. Analyses were nested by village of residence to account somewhat for unmeasured variables related to environmental exposure; however, there were undoubtedly other variables that may have influenced change in VBLL. The use of point-of-care testing equipment rather than ICPMS mildly decreased the accuracy of VBLLs. Our primary measure of DMSA effectiveness was ECP, which required an end-course VBLL within the defined timeframe, resulting in exclusion of five deaths during the first DMSA course, a possible source of bias. Another six deaths were in children with no courses meeting inclusion criteria. Exclusion of these deaths from 3,180 courses analysed is unlikely to have affected the primary outcome. To mitigate these exclusions, in our calculation of <2.5% mortality in severely poisoned children (initial VBLL ≥ 80 µg/dl) during the period studied we conservatively included all deaths considered caused or contributed to by lead poisoning (*n* = 11) in children who commenced any form of chelation.

### Conclusion

In this retrospective analysis of an emergency intervention, oral DMSA as a single agent was a pharmacodynamically effective chelator for children with severe lead poisoning, even when administered to obtunded encephalopathic children by nasogastric tube. A lower end-course VBLL was associated with higher pre-course VBLL, a longer interval between chelation courses, and chelation administered in the inpatient setting. The reduction in VBLL achieved by courses of oral DMSA chelation that were entirely or predominantly inpatient was comparable to that observed in previously published clinical trials of oral DMSA or IV CaNa_2_EDTA. Re-exposure to environmental lead contamination likely influenced the attenuated decline in VBLL associated with outpatient DMSA chelation. Decreased medication adherence is likely to have occurred in outpatient settings and may also have diminished the impact of chelation.

Although children with severe lead poisoning usually require multiple courses of DMSA chelation, the most desirable time interval between courses requires further investigation. This experience with basic supportive care and chelation in a large paediatric cohort adds significantly to the evidence base for clinical management of epidemic lead poisoning, particularly in resource-poor settings.

## Supporting Information

Figure S1 **Flowchart for actions based on blood lead level–capillary versus venous.**
(TIF)

Figure S2 **Flowchart for actions based on blood lead level—first treatment course.**
(TIF)

Figure S3 **Flowchart for actions based on blood lead level—second and subsequent treatment courses.**
(TIF)

Checklist S1 **STROBE checklist.**
(DOC)

## Abbreviations

ALT: alanine transaminase
BD: twice daily
CaNa_2_EDTA: calcium disodium versenate
CDC: US Centers for Disease Control and Prevention
DMSA: 2,3-dimercaptosuccinic acid
DOT: directly observed therapy
ICPMS: inductively coupled plasma mass spectrometry
IV: intravenous
ECP: end-course venous blood lead level as a percentage of pre-course venous blood lead level
MSF: Médecins Sans Frontières
TDS: thrice daily
VBLL: venous blood lead level
