# Supplementary figures and images for: Description of 3,180 Courses of Chelation with Dimercaptosuccinic Acid in Children ≤5 y with Severe Lead Poisoning in Zamfara, Northern Nigeria: A Retrospective Analysis of Programme Data
Source: PLoS Med. 2014 Oct 7;11(10):e1001739. doi: 10.1371/journal.pmed.1001739 (PMC4188566; doi:10.1371/journal.pmed.1001739)

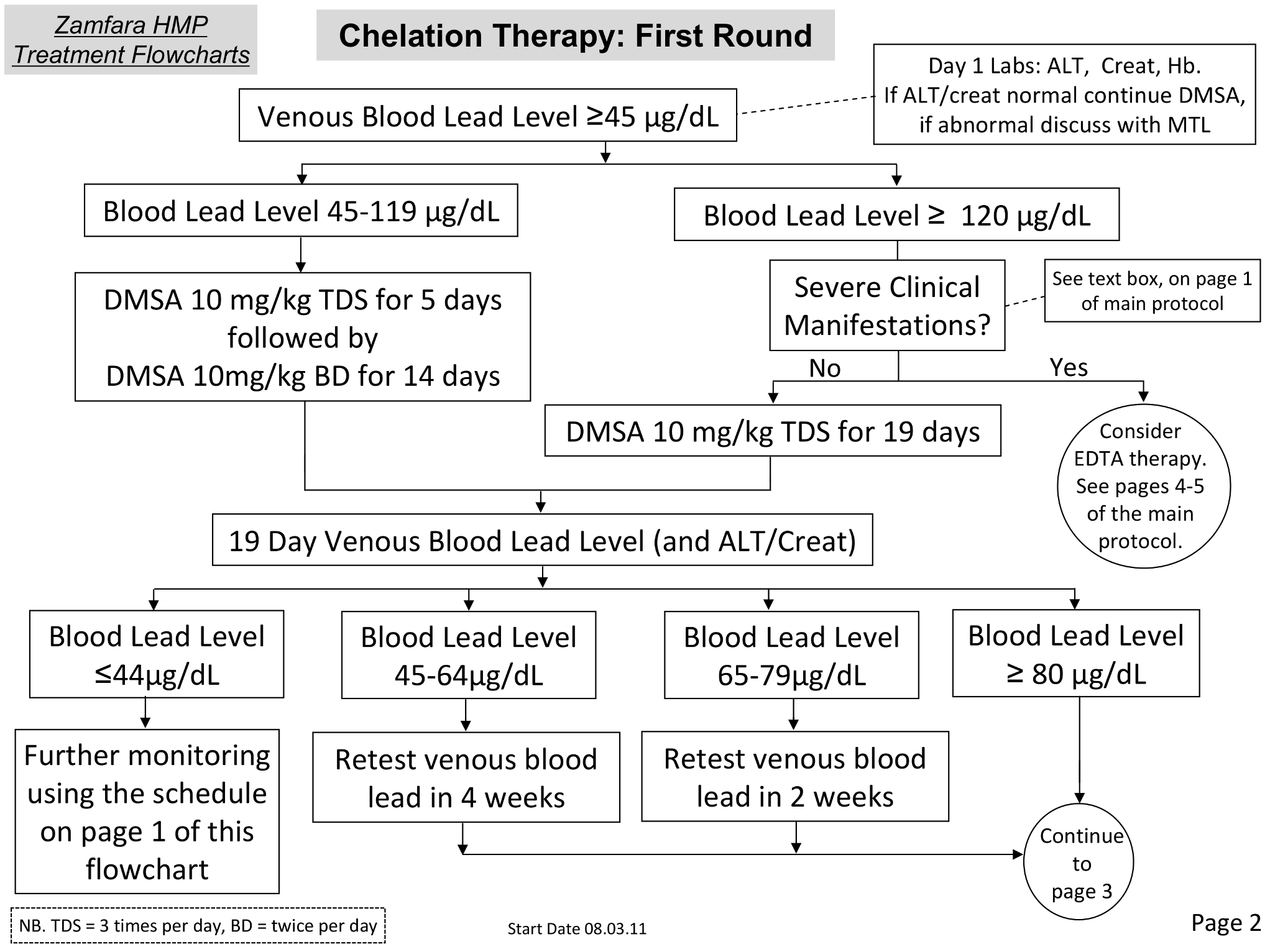

Supplement: Figure S2 — Flowchart for actions based on blood lead level—first treatment course. (TIF) [file pmed.1001739.s002.tif]

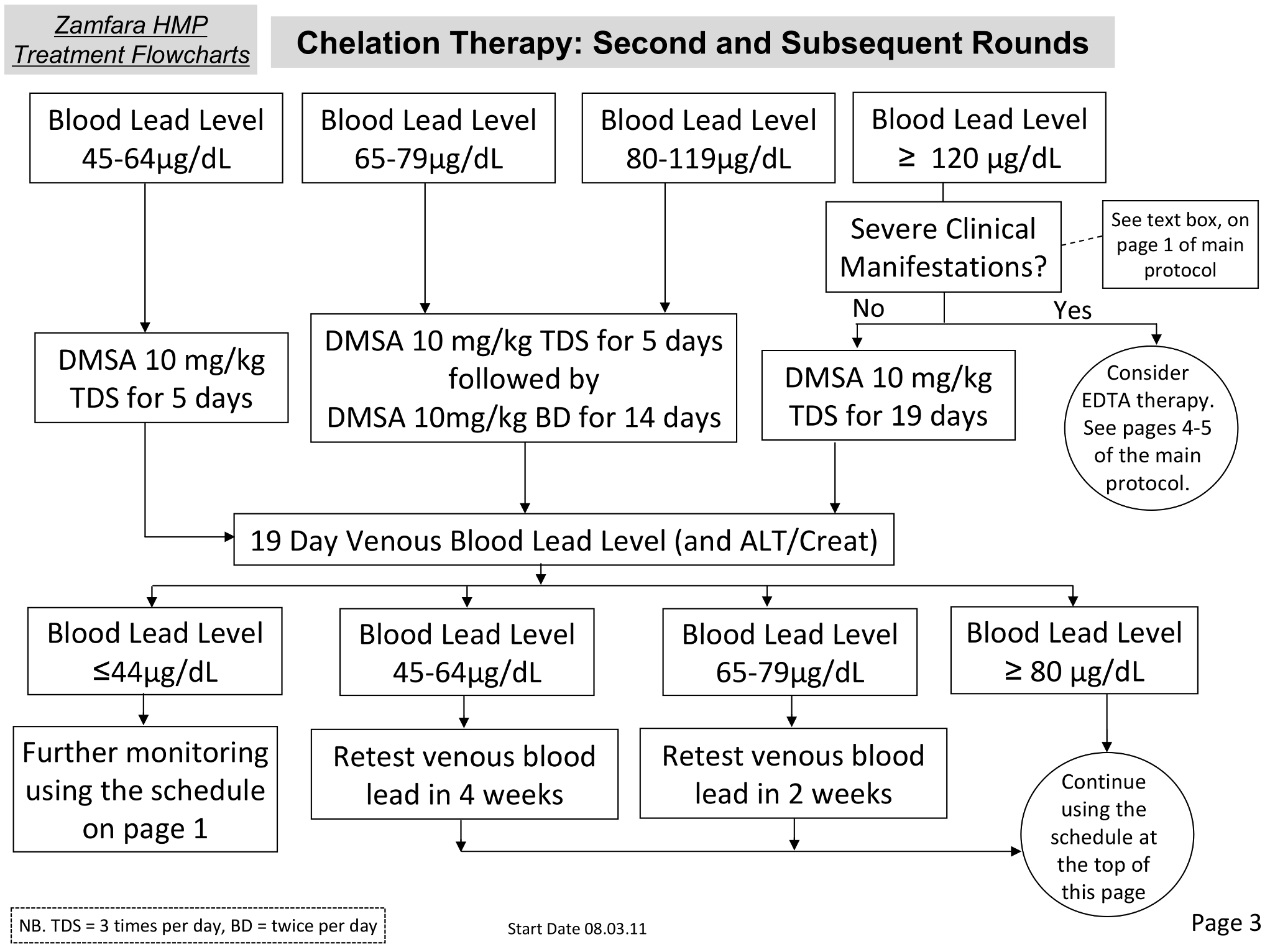

Supplement: Figure S3 — Flowchart for actions based on blood lead level—second and subsequent treatment courses. (TIF) [file pmed.1001739.s003.tif]
